# Reliability Issues of Mobile Nutrition Apps for Cardiovascular Disease Prevention: Comparative Study

**DOI:** 10.2196/54509

**Published:** 2024-09-04

**Authors:** Dang Khanh Ngan Ho, Wan-Chun Chiu, Jing-Wen Kao, Hsiang-Tung Tseng, Cheng-Yu Lin, Pin-Hsiang Huang, Yu-Ren Fang, Kuei-Hung Chen, Ting-Ying Su, Chia-Hui Yang, Chih-Yuan Yao, Hsiu-Yueh Su, Pin-Hui Wei, Jung-Su Chang

**Affiliations:** 1 School of Nutrition and Health Sciences, College of Nutrition, Taipei Medical University, 250 Wuxing Street, Xinyi District, Taipei, 110, Taiwan, 886 2-27361661 ext 6542; 2 Department of Nutrition, Wan Fang Hospital, Taipei Medical University, Taipei, Taiwan; 3 Department of Computer Science and Information Engineering, National Taiwan University of Science and Technology, Taipei, Taiwan; 4 Department of Dietetics, Taipei Medical University Hospital, Taipei, Taiwan; 5 Graduate Institute of Metabolism and Obesity Sciences, College of Nutrition, Taipei Medical University, Taipei, Taiwan; 6 Nutrition Research Center, Taipei Medical University Hospital, Taipei, Taiwan; 7 Chinese Taipei Society for the Study of Obesity (CTSSO), Taipei, Taiwan; 8 TMU Research Center for Digestive Medicine, Taipei Medical University, Taipei, Taiwan

**Keywords:** mobile apps, mHealth, dietary assessment, validity, cardiovascular disease prevention, app, apps, applications, application, nutrition, cardiovascular, nutrients, fitness, diet, mobile health

## Abstract

**Background:**

Controlling saturated fat and cholesterol intake is important for the prevention of cardiovascular diseases. Although the use of mobile diet-tracking apps has been increasing, the reliability of nutrition apps in tracking saturated fats and cholesterol across different nations remains underexplored.

**Objective:**

This study aimed to examine the reliability and consistency of nutrition apps focusing on saturated fat and cholesterol intake across different national contexts. The study focused on 3 key concerns: data omission, inconsistency (variability) of saturated fat and cholesterol values within an app, and the reliability of commercial apps across different national contexts.

**Methods:**

Nutrient data from 4 consumer-grade apps (COFIT, MyFitnessPal-Chinese, MyFitnessPal-English, and LoseIt!) and an academic app (Formosa FoodApp) were compared against 2 national reference databases (US Department of Agriculture [USDA]–Food and Nutrient Database for Dietary Studies [FNDDS] and Taiwan Food Composition Database [FCD]). Percentages of missing nutrients were recorded, and coefficients of variation were used to compute data inconsistencies. One-way ANOVAs were used to examine differences among apps, and paired 2-tailed *t* tests were used to compare the apps to national reference data. The reliability across different national contexts was investigated by comparing the Chinese and English versions of MyFitnessPal with the USDA-FNDDS and Taiwan FCD.

**Results:**

Across the 5 apps, 836 food codes from 42 items were analyzed. Four apps, including COFIT, MyFitnessPal-Chinese, MyFitnessPal-English, and LoseIt!, significantly underestimated saturated fats, with errors ranging from −13.8% to −40.3% (all *P*<.05). All apps underestimated cholesterol, with errors ranging from −26.3% to −60.3% (all *P<*.05). COFIT omitted 47% of saturated fat data, and MyFitnessPal-Chinese missed 62% of cholesterol data. The coefficients of variation of beef, chicken, and seafood ranged from 78% to 145%, from 74% to 112%, and from 97% to 124% across MyFitnessPal-Chinese, MyFitnessPal-English, and LoseIt!, respectively, indicating a high variability in saturated fats across different food groups. Similarly, cholesterol variability was consistently high in dairy (71%-118%) and prepackaged foods (84%-118%) across all selected apps. When examining the reliability of MyFitnessPal across different national contexts, errors in MyFitnessPal were consistent across different national FCDs (USDA-FNDSS and Taiwan FCD). Regardless of the FCDs used as a reference, these errors persisted to be statistically significant, indicating that the app’s core database is the source of the problems rather than just mismatches or variances in external FCDs.

**Conclusions:**

The findings reveal substantial inaccuracies and inconsistencies in diet-tracking apps’ reporting of saturated fats and cholesterol. These issues raise concerns for the effectiveness of using consumer-grade nutrition apps in cardiovascular disease prevention across different national contexts and within the apps themselves.

## Introduction

Cardiovascular diseases (CVDs) are leading causes of global mortality and major contributors to disability [1]. Excessive intake of saturated fats and cholesterol was significantly associated with increased risks of CVDs and all-cause mortality [2-4]. The American Heart Association has emphasized that reducing saturated fat intake in favor of unsaturated fats can significantly diminish CVD risks [5]. Managing these nutrients is crucial for early prevention efforts [6,7]. In this context, the advent of mobile diet-tracking apps may offer a modern solution for monitoring dietary saturated fats and cholesterol, with benefits such as accessibility, cost-effectiveness, and efficiency over traditional dietary assessment methods [8-10].

Khazen et al [11] has categorized diet-tracking apps into 2 main types: academic and commercial. Academic apps are developed with research-based validation but are often limited by their geographical scope and reliance on local food composition databases (FCDs). Some examples of academic apps are Australia’s Electronic Dietary Intake Assessment [12], Canada’s Keenoa [13], and Taiwan’s Formosa FoodApp [14]. On the other hand, commercial apps (such as MyFitnessPal, FatSecret, and Lose It!) are known for their extensive, international FCDs and are designed to cater to multilingual users [11,15,16]. They include a function that allows users to add new food products not available in the existing FCD, characterizing them as consumer-grade apps with a consumer-oriented approach. Language availability is another factor differentiating academic and consumer-grade apps [11]. Academic apps typically support only 1 or 2 languages and are tailored for specific research within certain populations, thus limiting their use in global studies [11,17]. In contrast, commercial apps, with their support for multiple languages [18], are aimed at a global audience, positioning them as versatile or “universal” tools accessible to users across the world.

The primary concerns with consumer-grade apps are the quality and reliability of their FCDs to estimate energy and nutrient intake [11,19]. Regarding the reliability, academic apps have been in agreement with conventional self-report dietary assessment methods such as 24-hour dietary recall [14,20,21]. In contrast, the accuracy of consumer-grade apps varies, generally showing a trend of underestimating nutritional values, with variability in accuracy across different commercial apps [19,22]. This is likely due to the varying quality of FCDs among commercial apps, which tend to suffer from missing or redundant data [19,22]. Although many studies have emphasized the disparities in dietary intake of total energy and macronutrients between data from commercial apps and national food databases [10,22,23], very few have delved into the variability in saturated fat and cholesterol values. While many commercial apps derive data from nutrition labels with mandated disclosures for saturated fats and cholesterol, these aspects in commercial nutrition apps remain insufficiently evaluated.

Currently, the reliability of the consumer-grade apps in tracking nutrients across different national standards remains underexplored. This study aimed to examine the reliability of mobile nutrition apps by focusing on saturated fat and cholesterol intake and the consistency of commercial apps across different national contexts. This critical evaluation focused on 3 key concerns: omission of saturated fat and cholesterol data, data consistency within an app, and the reliability of commercial apps across different national contexts.

## Methods

### App Selection

We selected widely used apps from 2 distinct regions, Taiwan and the United States, to represent linguistic and cultural differences. Specifically, we chose the academic app Formosa FoodApp [14] and the commercial app COFIT [24] given their widespread clinical and educational acceptance in Taiwan. In addition, we also explored 2 commercial US-based apps—MyFitnessPal and Lose It!—based on their broad acknowledgment, dominant presence in both clinical settings and among consumers, and citations in earlier literature [10,15,19]. The primary sources for nutrient data included the Taiwan FCD, US Department of Agriculture (USDA)–Food and Nutrient Database for Dietary Studies (FNDDS), and US food manufacturers and restaurants. Notably, MyFitnessPal and LoseIt! make user-generated food entries available to all users (Table 1).

**Table 1.** Description of the mobile app food composition databases (FCDs) in the study.

Characteristics	Formosa FoodApp	COFIT	MyFitnessPal-Chinese	MyFitnessPal-English	LoseIt!
Type of FCD	Academic	Commercial	Commercial	Commercial	Commercial
Manufacturer and country	Professor Susan Chang’s Lab (Taipei Medical University), Taiwan	Cofit Healthcare, Taiwan	Francisco Partners, United States	Francisco Partners, United States	FitNow, United States
FCD language	Chinese	Chinese	Chinese (added by users)	English	English
FCD sources	Taiwan FCD, USDA^a^-FNDSS^b^, Vietnam FCD, Indonesia FCD, food manufacturers, and restaurants	Taiwan FCD, food manufacturers, and restaurants	Taiwan FCD and users	USDA-FNDSS, food manufacturers, restaurants, and users	USDA-FNDSS, food manufacturers, restaurants, and users
User-added function	No	No	Yes	Yes	Yes

a USDA: US Department of Agriculture.

b FNDSS: Food and Nutrient Database for Dietary Studies.

### Food Items and Code Selection

**Figure 1.**
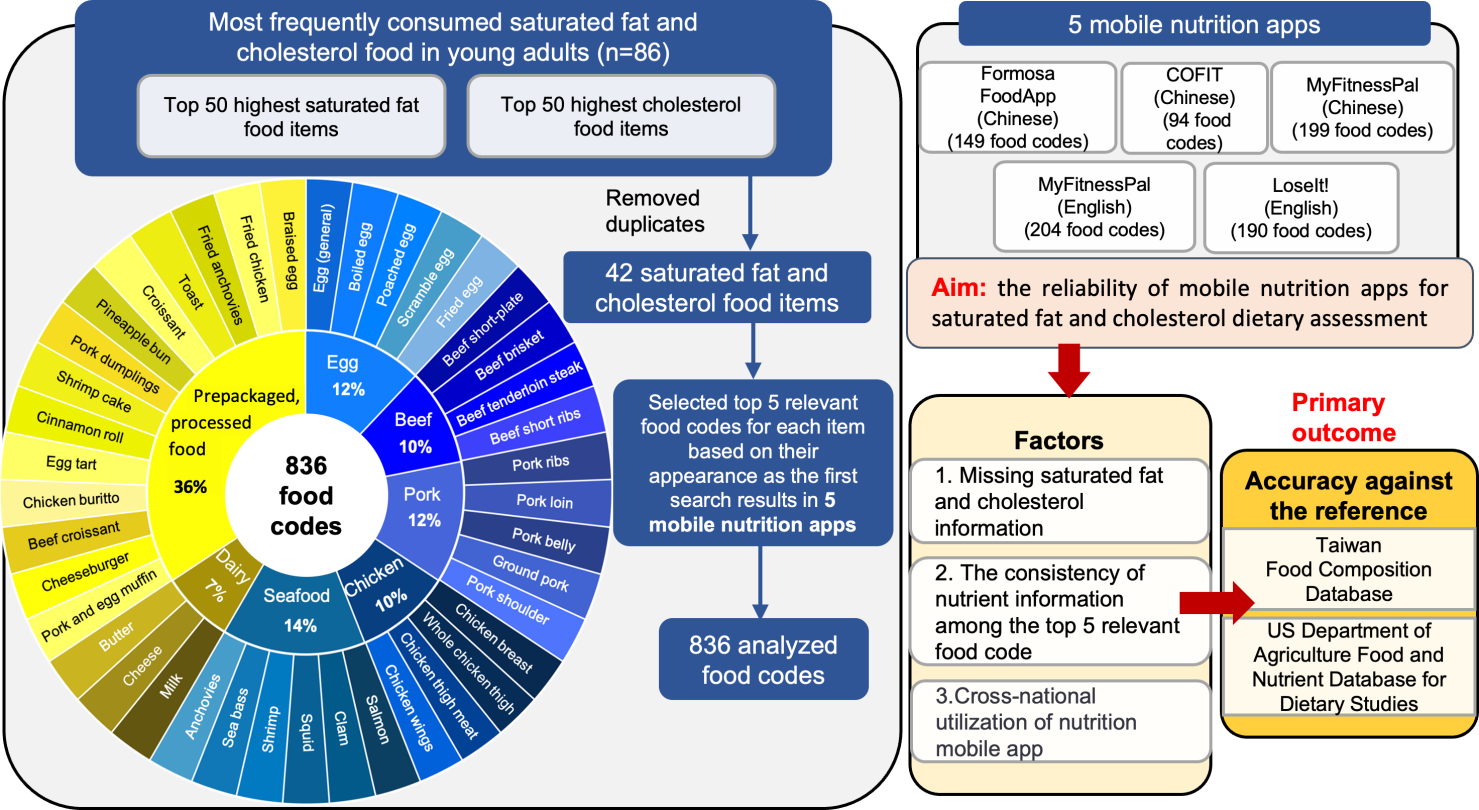
Flowchart of the selection process for food items and food codes. Food items included are those that contribute the most to cholesterol and saturated fat intake levels, ranked based on findings from our previous Formosa FoodApp validation study [14].

Figure 1 shows the flowchart of the food item and food code selection process. Briefly, data from our Formosa FoodApp validation study were used for the analyses [14], which involved 86 healthy adults aged 19-26 years who tracked their daily diet using the Formosa FoodApp. To identify food items that contributed to high cholesterol and saturated fat intake, a nutrition-trained investigator ranked frequently consumed, saturated fat– and cholesterol-containing items from our prior study. The initial assessment generated 2 lists of 50 food items each. After eliminating duplicates and similar entries, we finalized a list comprising 42 unique food items.

To mitigate potential biases from initially using data from the Formosa FoodApp, which may predominantly reflect Taiwanese dietary preferences, we verified the availability of these 42 food items across all apps selected for this study. The comprehensive list of these food items is provided in Multimedia Appendix 1 for reference. We further grouped these 42 food items into broader categories—such as eggs, beef, pork, chicken, dairy, seafood, and prepackaged and processed foods—to enhance the representativeness of our analysis across diverse national diets.

Food items were entered using the search feature for the free versions of Formosa FoodApp, COFIT, MyFitnessPal, and Lose It!. Searches were performed across Formosa FoodApp and COFIT in Chinese and LoseIt! in English. To explore MyFitnessPal’s reliability across different national contexts, searches were conducted in both Chinese and English, and we compared the errors in nutrient data from its English and Chinese versions. To ensure a clear distinction between MyFitnessPal in Chinese and English, we refer to them as MyFitnessPal-Chinese and MyFitnessPal-English, respectively, throughout the manuscript. Given the vast array of food codes that appeared in the search results and to make the analysis more manageable and representative, we selected the top 5 relevant food codes that appeared first in the search results for each item as representative subsets. Energy contents and nutrients including total carbohydrate, protein, total fat, saturated fat, and cholesterol values were extracted from the apps (Figure 1). The selection process underwent a review and confirmation by a second investigator. Portion sizes for the selected food codes were standardized at 100 g across all databases. Data from all selected apps and reference databases were documented for each food code and entered into a Microsoft Excel dataset.

### Data and Statistical Analysis

We calculated the mean percentage error for energy, total carbohydrate, protein, total fat, saturated fat, and cholesterol values as the difference between the nutrient intake values from the apps and the corresponding values from the reference databases, expressed as a percentage error. This error, calculated for each food code in each app, is given by the following:


$$Percentage\;error\;\left(\%\right)=\frac{App\;nutrient\;value-Reference\;nutrient\;value}{Reference\;nutrient\;value}\times 100$$

We focused on 2 key components of variability for saturated fats and cholesterol: missing nutrient data and inconsistencies of nutrient data for the same food item across 5 codes. Missing nutrient information for each food item was determined as the ratio of missing nutrient information to the total selected food codes for that item, presented as a percentage:


$$Percentage\;of\;missing\;nutrient\;data\;\left(\%\right)=\frac{Number\;of\;missing\;nutrient\;data\;points}{Total\;number\;of\;selected\;food\;codes\;for\;a\;food\;item}\times 100$$

We calculated coefficients of variation (CVs) to address nutrient data inconsistencies across 5 food codes for the same item within an app. CVs were obtained by taking the square root of the average reporting variance within a food item, divided by the mean of the items (app values over food codes). A CV was calculated for each food item, and then mean CVs were determined based on the food group and app. Our analysis involved paired 2-tailed *t* tests for app-to-reference comparisons (paired within food codes) and a 1-way ANOVA for app-to-app variations. A linear regression analysis was used to assess the factor associated with the percentage of underestimated error of nutrients compared to reference databases. To assess the reliability of a commercial app universally used in different national contexts, we conducted a cross-national analysis, comparing errors in both MyFitnessPal-Chinese and MyFitnessPal-English against the USDA-FNDDS and Taiwan FCD reference databases. All analyses were performed in SPSS (version 23; IBM Corp) and GraphPad Prism 8 (GraphPad Software), with *P* values of <.05 considered statistically significant.

### Ethical Considerations

This study conducted a secondary analysis of data previously collected in an earlier study [14], which received approval from the Taipei Medical University Institutional Review Board (N202101046). Written informed consent was obtained from all participants. The data used in this analysis were deidentified.

## Results

### Characteristics of Mobile Nutrition Apps

Among the selected nutrition apps, Formosa FoodApp is an academic mobile nutrition app, while COFIT, MyFitnessPal-Chinese, MyFitnessPal-English, and LoseIt! are all commercial apps (Table 1). Figure 1 details the distribution of 42 food items across 7 main food categories. The dominant food groups were prepackaged processed foods (15 items), seafood (6 items), and pork and egg (5 items each).

### Reliability of Mobile Nutrition Apps Across Different National Standards

**Figure 2.**
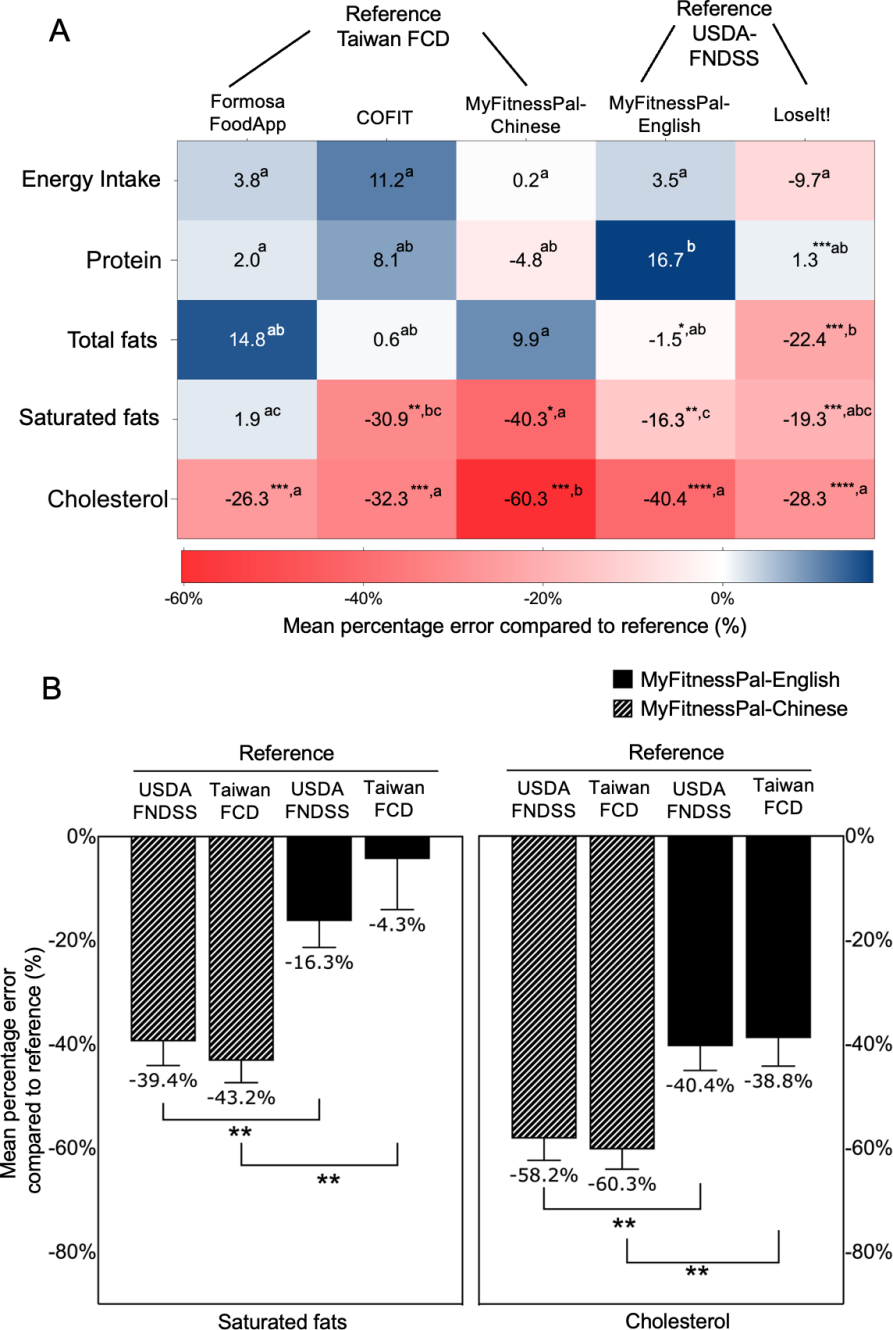
(A) Heat map of mean percentage errors of nutrients across mobile apps compared to reference databases. Different letters (a, b, ab, c, bc, and abc) indicate statistically significant differences in mean percentage errors among groups based on 1-way ANOVA (*P*<.05). a: statistically different from b, bc, and c; b: statistically different from a, ab, and c; ab: not significantly different from a or b; c: statistically different from a, ab, and b; bc: not significantly different from b or c, but different from a; abc: not significantly different from a, b, or c. Asterisks (*) denote the level of statistical significance in paired 2-tailed *t* tests between the app and the reference database: *P<.05, **P<.01, ***P<.001, and ****P<.0001. (B) Cross-national comparison of percentage errors in saturated fats and cholesterol between the English and Chinese platforms of MyFitnessPal. **P<.01 by a 1-way ANOVA indicating differences in percentage errors among groups. FCD: Food Composition Database; FNDDS: Food and Nutrient Database for Dietary Studies; USDA: US Department of Agriculture.

Figure 2A presents the mean percentage errors of app nutrient data against national reference databases. Most apps showed no differences in energy or macronutrients compared to their respective national references, with the exception of LoseIt!, which underestimated protein by −1.3% and fats by −22.4% compared to the USDA-FDNSS. COFIT, MyFitnessPal (in both languages), and LoseIt! underestimated saturated fats with errors ranging from −16.3% to −40.3% (all *P*<.05). All apps underestimated cholesterol, with errors ranging from −26.3% to −60.3% (all *P*<.05).

Figure 2B displays the mean percentage errors in MyFitnessPal-Chinese and MyFitnessPal-English using the USDA-FNDSS and Taiwan FCD. The Chinese version underestimated saturated fats by around −40% (−43.2% for the Taiwan FCD and −39.4% for the USDA-FNDSS) and cholesterol by −60% (−58.2% for the USDA-FNDSS and −60.3% for the Taiwan FCD). In contrast, the English version showed −10% errors for saturated fat (−16.8% for the USDA-FNDSS and −4.3% for the Taiwan FCD) and −40% for cholesterol (−40.4% for the USDA-FNDSS and −38.8% for the Taiwan FCD). Notably, these discrepancies, significant at *P*<.01 for both nutrients, remained unchanged regardless of the reference database used.

### Omissions of Saturated Fats and Cholesterol

**Figure 3.**
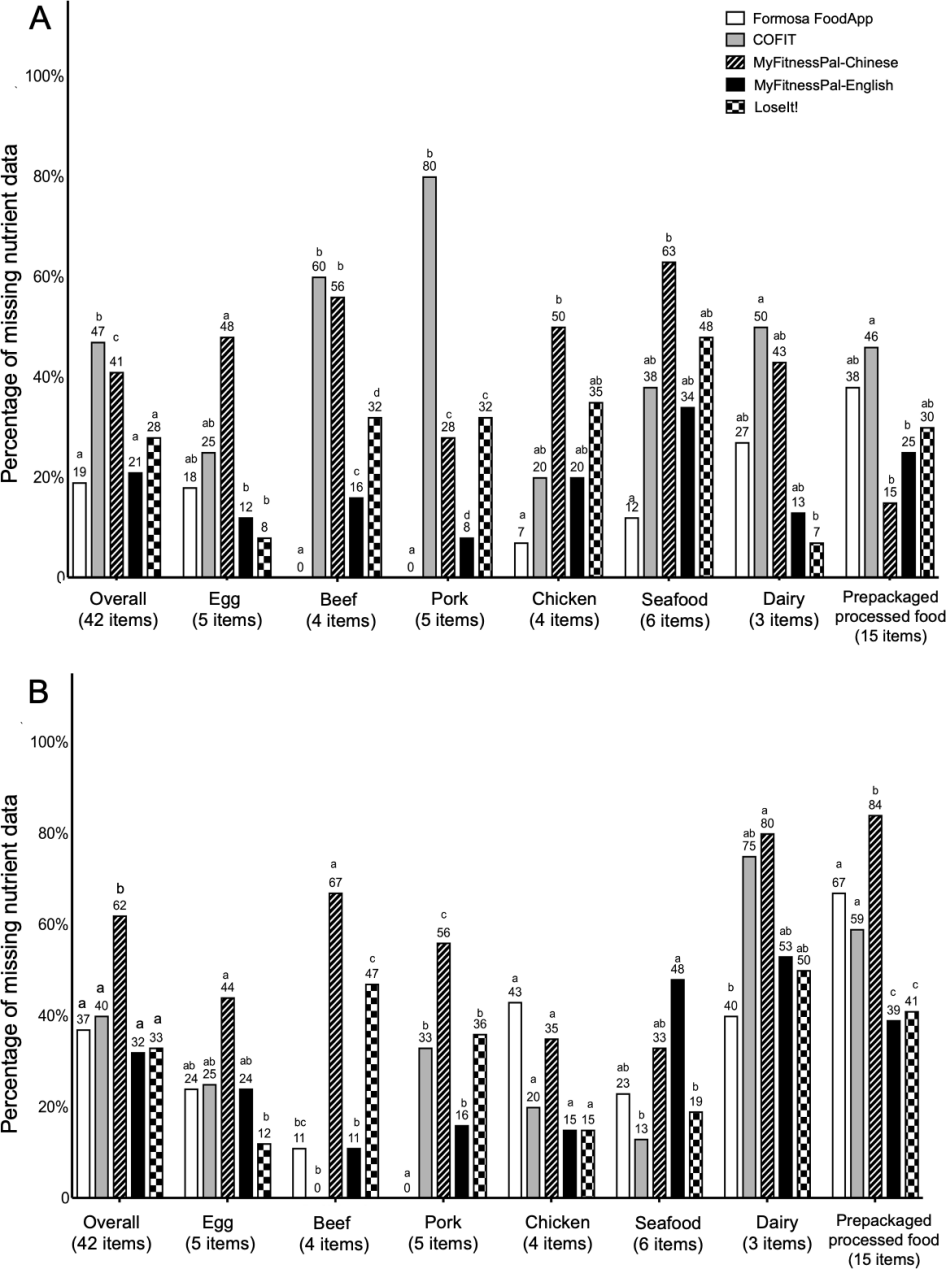
Omission rates (percentage of missing nutrient information) for (A) saturated fats and (B) cholesterol across the mobile nutrition apps. Different letters (a, b, c, d, ab, and bc) indicate a significant *P* value (*P*<.05) for Fisher test comparing differences in omission rates between the apps among the mobile app food databases. a: statistically different from b, bc, c, and d; b: statistically different from a, ab, c, and d; ab: not significantly different from a or b; c: statistically different from a, ab, b, and d; d: statistically different from a, b, and c; bc: not significantly different from b or c, but different from a and d.

Omission rates for different nutrients considerably varied. Total fats exhibited the lowest omission range, spanning from 0% to 21%. In comparison, saturated fats and cholesterol showed higher rates of omission, ranging from 19% to 47% and from 21% to 62%, respectively (data not shown). COFIT showed the highest missing information for saturated fats at 47% (Figure 3A), while MyFitnessPal-Chinese recorded the highest omission at 62% for cholesterol (Figure 3B).

For saturated fats, both COFIT and MyFitnessPal-Chinese showed substantial omissions, especially for beef (60% and 56%, respectively), pork (80% and 28%, respectively), and dairy (50% and 43%, respectively). For cholesterol, there were significant differences in omission rates between MyFitnessPal-Chinese and MyFitnessPal-English (62% vs 32%; *P*<.05), notably for beef (67% vs 11%), pork (56% vs 16%), dairy (80% vs 53%), and prepackaged processed foods (84% vs 39%).

### Variability Extents of Saturated Fat and Cholesterol

**Figure 4.**
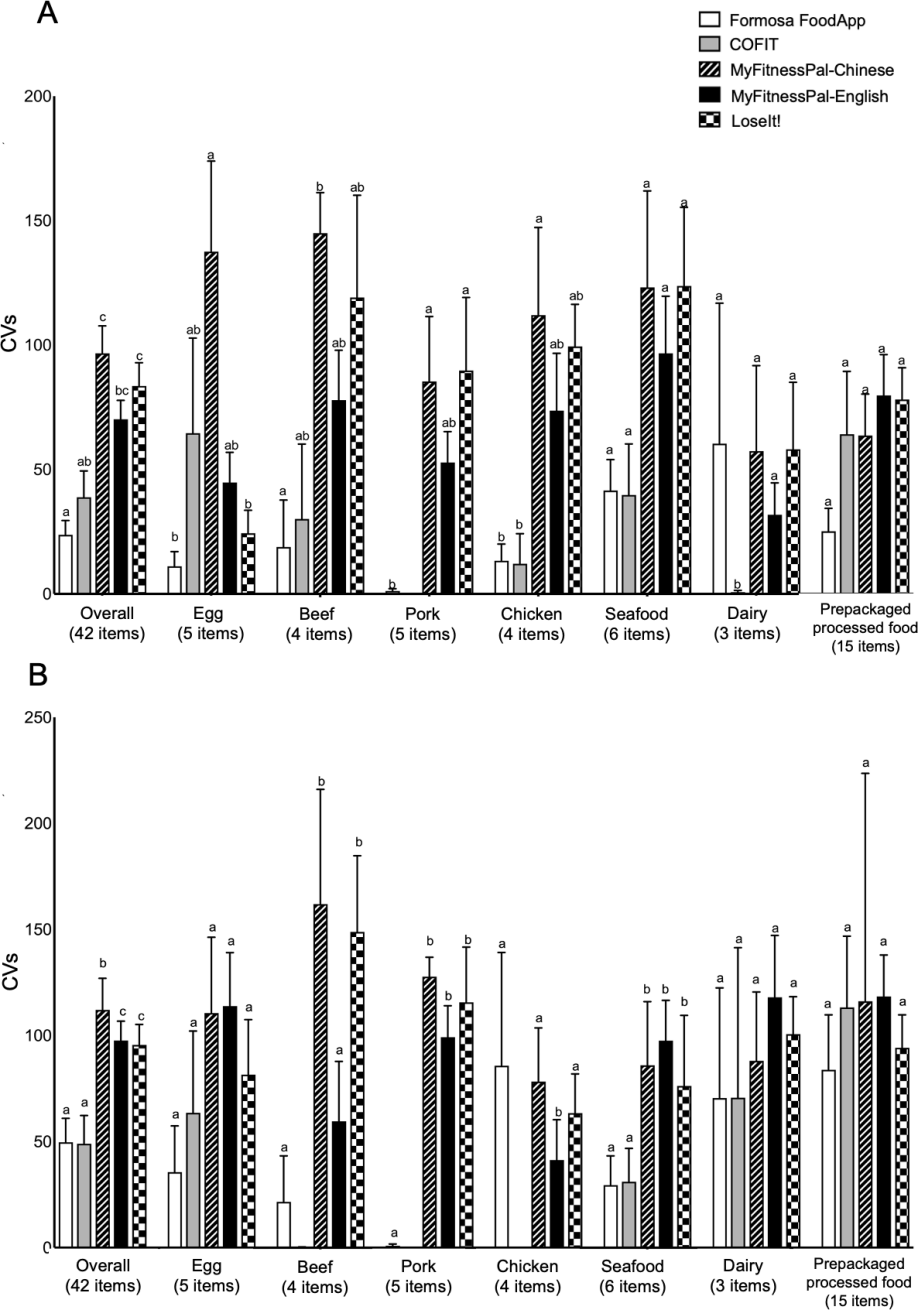
Mean coefficient of variations (CVs) for (A) saturated fats and (B) cholesterol, calculated as the percentage ratio of the SD to the mean, for 42 food items grouped by food groups across the apps. Different letters (a, b, ab, and c) indicate a significant *P* value (*P*<.05) for 1-way ANOVA comparing differences in the CVs among the apps within food groups. a: statistically different from b and c; b: statistically different from a, ab, and c; ab: not significantly different from a or b; c: statistically different from a, ab, and b.

We calculated CVs to address saturated fat and cholesterol data consistencies, by assessing 5 food codes for identical items within a given app (Figure 4). For saturated fats, the lowest mean CVs were observed in Formosa FoodApp (23.8%) and COFIT (38.9%). The highest mean CVs were observed in MyFitnessPal-Chinese (96.7%) and LoseIt! (83.6%). A similar trend was also observed for cholesterol.

Saturated fat variability was high for beef (78%-145%), chicken (74%-112%), and seafood (97%-124%) among MyFitnessPal-Chinese, MyFitnessPal-English, and LoseIt!, respectively. Cholesterol variability was high for dairy (71%-118%) and prepackaged foods across all apps (84%-118%; Figure 4).

## Discussion

### Principal Findings

To the best of our knowledge, this is the first study to critically examine the reliability of mobile nutrition apps’ reported data on saturated fats and cholesterol against 2 different national FCDs. We found significant errors up to −40.3% for saturated fats and −60.3% for cholesterol, suggesting that these apps may provide unreliable data when assessing diets, particularly in the context of CVD prevention. These findings align with the findings by Shinozaki and Murakami [25] on MyFitnessPal’s nutrient underestimation of saturated fats and cholesterol (85.1% and 97.6%, respectively). Similarly, Siniarski et al [16] also reported errors up to 57.3% in mobile app predictions for saturated fats compared to Polish reference data. Furthermore, our results demonstrate that errors in MyFitnessPal’s nutrient reporting are consistent across different national FCDs (USDA-FNDDS and Taiwan FCD). Such errors remained statistically significant, regardless of the FCDs used as a reference. This problem suggests that the errors are not merely due to mismatches or variations in external FCDs but are rooted in the app’s core database.

Data inconsistencies within consumer-grade apps, evidenced by significant omissions and variability, compromise their reliability. For instance, saturated fat variability was notably high across different food groups, with beef ranging from 78% to 145%, chicken from 74% to 112%, and seafood from 97% to 124% among MyFitnessPal-Chinese, MyFitnessPal-English, and LoseIt!, respectively. Similarly, cholesterol variability was consistently high in dairy (71%-118%) and prepackaged foods (84%-118%) across all apps. COFIT lacked almost 50% of its saturated fat data, and MyFitnessPal-Chinese was missing over 60% of its cholesterol information. This is concerning as food labeling regulations require clear disclosure of these nutrients [6], yet many entries incorrectly list these values as zero, deviating from standard guidelines [26]. This issue is even more pressing in the context of prepackaged foods. Previous research from our team emphasized this challenge, revealing that data omissions related to prepackaged foods significantly impact the reliability of mobile dietary assessments [14]. This emphasizes the urgency for databases to stay updated with new market introductions.

The lack of transparency in food sourcing within consumer-grade apps, particularly when combined with the extensive volume of FCDs [11,27], can significantly confuse users during food logging [28,29]. As consumer-grade apps allow for a vast array of food items to be listed—often without clear sourcing information, users face the daunting task of navigating through numerous results to find the most accurate match for their consumed foods. The entries frequently do not specify their nutritional data sources. Our data showed that only a fraction of the analyzed food codes transparently disclosed their sources. Specifically, just 51.5% (n=105) of MyFitnessPal-English’s 204 food codes and 43.7% (n=87) of its Chinese counterpart’s 199 food codes provided clear sourcing information. Meanwhile, LoseIt! transparently sourced only 37.4% (n=71) of its 190 codes. Formosa FoodApp led with 78.2% (n=115) for its 147 codes, while COFIT lagged at 61% (n=57) for its 94 codes. This lack of clarity can lead to confusion and inaccuracies, underscoring the need for improved transparency in food sourcing to facilitate more precise dietary tracking.

Our analysis also delved into the complexities of maintaining accuracy within multilingual universal apps, using MyFitnessPal as a case study. Although errors in MyFitnessPal’s nutrient reporting are consistent across different national FCDs (USDA-FNDDS and Taiwan FCD), it is important to note that the larger errors observed in MyFitnessPal-Chinese, as opposed to the English version, highlight another challenge for universal apps: ensuring uniform accuracy across different languages and, by extension, cultural contexts. Several factors may contribute to the discrepancies observed in MyFitnessPal. First, the integration of multiple FCDs combined with user-generated content without stringent quality control can introduce variability in nutrient data [27]. Second, nutritional composition can also vary by region due to differences in food production, processing, and preparation practices, further complicating accurate nutrient estimation [30]. Lastly, user-generated content can exacerbate inaccuracies, as this content may not undergo rigorous verification processes, leading to inconsistencies in nutrient reporting [27]. Together, these factors highlight the complexities of providing accurate, universal dietary tracking tools and the critical need for app developers to address these challenges to enhance the reliability and global applicability of their platforms.

This study has highlighted significant errors and inconsistencies in various consumer-grade apps. Academic apps such as Formosa FoodApp, which show fewer reporting errors and greater transparency in nutrient sourcing, are crucial for health professionals who require precise and validated data to make evidence-based dietary recommendations, particularly for CVD prevention. However, these apps encounter financial and regional limitations that restrict their usability. As academic apps are usually funded by research grants or academic institutions for specific purposes, their development and maintenance are often constrained, limiting updates and expansion. Moreover, many academic apps are tailored for specific regional diets, such as the Taiwanese focus of Formosa FoodApp, which narrows their international relevance. Additionally, these apps often lack engaging user interfaces and features that are essential for widespread user adoption [14]. Innovative funding models supporting extensive food databases and multilingual capabilities are needed to make these tools globally accessible and competitive with commercial apps. Khazen et al [11] suggested that an ideal dietary tracking tool would combine the features of commercial apps with the accuracy of academic apps, increasing their utility in global research and public health initiatives.

Our study contributed to the current literature by critically examining how accurately mobile apps can estimate the intake of saturated fats and cholesterol in different national contexts. Additionally, by investigating the consistency of data within and across the apps, we have highlighted several challenges these apps face in providing reliable nutritional information in the context of CVD prevention. Nonetheless, our methodology has inherent limitations. The data from a young demographic may introduce bias, potentially limiting the applicability of findings across all age groups. By focusing on only the top 5 food codes per item, we might not have captured the full extent of the data variability within each app. This strategy, although deliberate, possibly overlooked broader inaccuracies or consistencies in less frequently selected food items. Our cross-national examination predominantly relied on MyFitnessPal’s performance, potentially narrowing the applicability of our findings to other multilingual apps. Furthermore, the discrepancy in the number of selected food codes across apps—with MyFitnessPal at 204 and COFIT at 94, for instance—introduces bias when comparing results across platforms. Additionally, we did not factor in user behaviors such as app update frequencies, which can intermittently influence data accuracy. The evolving nature of these apps, with ongoing food item additions and updates by developers and users alike, suggests that database accuracy might shift over time. As the landscape of mobile health apps is dynamic, future studies would benefit from broader sampling and recognizing of these continual database evolutions, ensuring a more encompassing perspective on app reliability.

### Conclusions

Our study reveals substantial inaccuracies and inconsistencies in mobile nutrition apps’ reporting of saturated fats and cholesterol, highlighting challenges in ensuring data reliability across different national contexts and within the apps themselves. As digital tools are increasingly incorporated in CVD prevention and care, rigorous assessments and continuous refinements are crucial to ensure that they serve as dependable resources for both professionals and the public.

## Supplementary material

10.2196/54509 Multimedia Appendix 1 Description of mobile app databases and analyzed food items in the study.

## Abbreviations

CV: coefficient of variation
CVD: cardiovascular disease
FCD: food composition database
FNDDS: Food and Nutrient Database for Dietary Studies
USDA: US Department of Agriculture
