# Supplementary material for: Reliability Issues of Mobile Nutrition Apps for Cardiovascular Disease Prevention: Comparative Study
Source: JMIR Mhealth Uhealth. 2024 Sep 4;12:e54509. doi: 10.2196/54509 (PMC11391091; doi:10.2196/54509)
Supplement: Multimedia Appendix 1 [file mhealth-v12-e54509-s001.docx]

**Multimedia Appendix 1.** Description of mobile app databases and analyzed food items in the study.

| Characteristic | Formosa FoodAPP – Chinese | COFIT-Chinese | MyFitnessPal (Chinese) | MyFitnessPal (English) | LoseIt! (English) |
| --- | --- | --- | --- | --- | --- |
| Type of Food Composition Database (FCD) | Academic | Commercial | Commercial | | Commercial |
| Manufacturer, country | Professor  Susan Chang Lab  (Taipei Medical University)  Taiwan | Cofit Healthcare Inc., Taiwan | Francisco Partners., United States | | FitNow Inc., United States |
| FCD language | Chinese | Chinese | Chinese (added by users) | English | English |
| FCD sources | Taiwan FCD, USDA FNDSS, Vietnam FCD, Indonesia FCD, food manufacturers and restaurants | Taiwan FCD, food manufacturers and restaurants | Taiwan FCD, users | USDA FNDSS, food manufacturers and restaurants, users | USDA FNDSS, food manufacturers and restaurants, users |
| User-added function | Yes | No | yes | | yes |
| Details of 42 analyzed food items | 雞蛋平均值, 雞水煮蛋, 荷包蛋, 炒蛋, 煎蛋, 牛五花肉火鍋片, 牛前胸肉, 帶骨牛小排, 腓力牛排, 豬小排, 豬大里肌, 豬去皮腹脇肉, 豬絞肉, 豬上肩肉, 去皮去骨雞腿, 清腿平均值, 去皮清肉, 翅腿, 大西洋鮭魚, 草蝦仁, 日本花鱸平均值, 文蛤, 台灣鎖管, 魩仔魚, 全脂鮮乳, 切片乾酪, 奶油, 土司, 冷凍豬肉水餃, 可頌, 菠蘿麵包, 冷凍雞塊, 蛋塔, 冷凍蝦餅, 小魚干, 豬肉滿福堡加蛋, 花生培根牛肉堡, 肉桂捲, 嫩雞沙拉捲餅, 滷蛋平均值, 炸雞腿排, 起司牛肉可頌 | | | Egg , Boiled egg, Poached egg, Fried egg, Scrambled egg, Beef short plate, Beef brisket, Beef tenderloin steak, Beef short ribs, Pork ribs, Pork loin, Pork belly, Ground pork, Pork shoulder, Chicken thighs meat, Chicken thighs with bone, Chicken breast, Chicken wings, Salmon, Shrimp, Sea bass, Clam, Small squid, Anchovies, Fresh milk, Cheese, Butter, Toast, Pork dumplings, Croissant, Pineapple bun, Chicken nuggets, Egg tart, Shrimp cake, Small dried fish, Muffin (pork and egg), Burger King cheeseburger, Cinnamon roll, Tender chicken burrito, Braised egg, Fried chicken, Beef croissant | |
| Total analyzed food codes | 149/836 (18%) | 94/836 (11%) | 199/836 (24%) | 204/836 (24%) | 190/836 (23%) |
| Total food items < 3 food codes | 22/42 (21%) | 23/42 (62%) | 10/42 (5%) | 21/42 (0%) | 22/42 (10%) |

FCD, Taiwan Food Composition Database

USDA FNDDS, US Department of Agriculture Food and Nutrient Database for Dietary Studies
